# Supplementary material for: Neuronavigated Cerebellar 50 Hz tACS: Attenuation of Stimulation Effects by Motor Sequence Learning
Source: Biomedicines. 2023 Aug 8;11(8):2218. doi: 10.3390/biomedicines11082218 (PMC10452137; doi:10.3390/biomedicines11082218)
Supplement: Supplementary file 1 [file biomedicines-11-02218-s001.zip › biomedicines-2507140-Supplementary Figure.pdf]

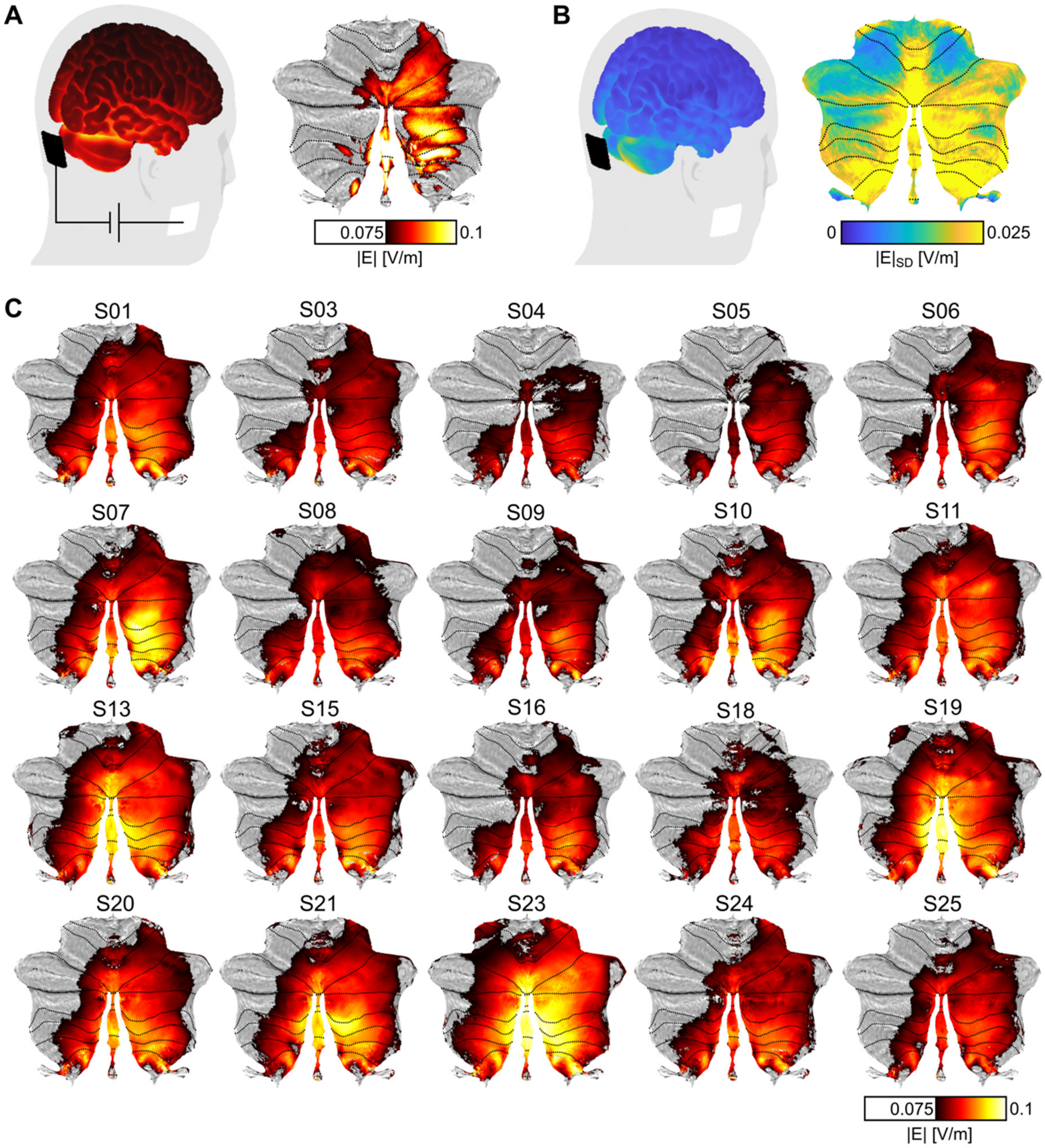

Figure S1. Individual electric field magnitudes in the cerebellum. Average (**A**) and standard deviation (SD) (**B**) of electric field magnitude for the complete sample were interpolated on cortical and cerebellar surfaces of the ICBM152, as well as on a cerebellar flatmap. (**C**) Individual electric fields were interpolated on a cerebellar flatmap. The variability across subjects mainly affects the extent of the electric field to the not targeted left hemisphere and the overall intensity.
