# Supplementary material for: Neuronavigated Cerebellar 50 Hz tACS: Attenuation of Stimulation Effects by Motor Sequence Learning
Source: Biomedicines. 2023 Aug 8;11(8):2218. doi: 10.3390/biomedicines11082218 (PMC10452137; doi:10.3390/biomedicines11082218)
Supplement: Supplementary file 1 [file biomedicines-11-02218-s001.zip › biomedicines-2507140-Supplementary Tables.pdf]

Supplementary Table S1. Mean stimulator output to evoke an MEP of 1 mV ± SEM

	s-tACS$_{Rest}$	s-tACS$_{MSL}$	l-tACS$_{Rest}$	l-tACS$_{MSL}$
stimulator output (in %)	51.15 ± 2.66	50.85 ± 2.33	50.55 ± 2.54	51.6 ± 2.65

Supplementary Table S2. Descriptive data regarding AMT, mean ± SEM

	s-tACS$_{Rest}$	s-tACS$_{MSL}$	l-tACS$_{Rest}$	l-tACS$_{MSL}$
pre (in %)	42.20 ± 1.04	44.40 ± 1.46	42.75 ± 1.37	42.95 ± 1.14
post 1 (in %)	41.70± 1.00	43.60 ± 1.54	42.80 ± 1.36	42.25 ± 1.15
post 2 (in %)	42.00 ± 1.00	44.00 ± 1.52	42.10 ± 1.31	41.20 ± 1.25

Supplementary Table S3. Descriptive data regarding RMT, mean ± SEM

	s-tACS$_{Rest}$	s-tACS$_{MSL}$	l-tACS$_{Rest}$	l-tACS$_{MSL}$
pre (in %)	49.53 ± 2.35	48.25 ± 2.22	49.19 ± 1.92	47.59 ± 1.94
post 1 (in %)	48.82 ± 2.18	48.88 ± 2.41	49.44 ± 2.17	48.12 ± 2.00

Supplementary Table S4. Descriptive electric field magnitude, bias and spatial extent with respect to nine lateralized cerebellar ROIs. Means ± standard deviations are shown for the left (L) and right (R) cerebellar hemisphere, respectively.

ROI	Hemisphere	$\|E\|_{target}$ [V/m]	$\|E\|_{bias}$ [mm]	$\|E\|_{extent}$ [mm]
**III**	L	0.09 ± 0.008	27 ± 5.4	35 ± 1.5
	R	0.07 ± 0.008	25 ± 4.6	33± 1.4
**IV/V**	L	0.08 ± 0.009	33 ± 5.6	37± 1.4
	R	0.09 ± 0.008	26 ± 4	33± 1.2
**VI**	L	0.09 ± 0.011	33 ± 6.8	39± 1.4
	R	0.1 ± 0.01	24 ± 2.6	34± 0.6
**Crus I**	L	0.08 ± 0.013	38 ± 5.8	43 ± 1.5
	R	0.11 ± 0.013	24 ± 4.4	36 ± 0.8
**Crus II**	L	0.09 ± 0.017	33 ± 5.4	42 ± 1.5
	R	0.11 ± 0.017	20 ± 5.1	36 ± 1
**VIIb**	L	0.1 ± 0.018	33 ± 3.3	42 ± 1.2
	R	0.12 ± 0.016	22 ± 7.4	37 ± 1.2
**VIII**	L	0.11 ± 0.014	26 ± 2	40 ± 1
	R	0.12 ± 0.01	18 ± 6	36 ± 1.4
**IX**	L	0.12 ± 0.008	16 ± 3.3	36 ± 1
	R	0.13 ± 0.009	12 ± 4.1	35 ± 1.2
**X**	L	0.07 ± 0.008	27 ± 1.9	39 ± 0.8
	R	0.09 ± 0.011	21 ± 7.6	35 ± 1.4

Supplementary Table S5. Follow-up *t*-tests assessing electric field magnitude and bias differences across cerebellar ROIs in the right (R) hemisphere. *T*-value, *p*-value and Cohens *d* is depicted for every pairwise *t*-tests. Asterisks indicate significant results (Bonferroni-corrected).

	$\|E\|_{target}$			$\|E\|_{bias}$		
**Test conditions (R)**	t_{19}	*p*	*|d|*	t_{19}	*p*	*|d|*
**Crus I VS Crus II**	-2.4	> .9	0.54	3.4	.108	0.76
**Crus I VS III**	*8.7**	*< .001*	*2*	-0.62	> .9	0.14
**Crus I VS IV/V**	*7.6**	*< .001*	*1.7*	-1.03	> .9	0.23
**Crus I VS VI**	3.4	.104	0.76	0.59	> .9	0.13
**Crus I VS VIIb**	*-4.7**	*.005*	*1.06*	1.31	> .9	0.29
**Crus I VS VIII**	*-7.2**	*< .001*	*1.61*	*4.07**	*.024*	*0.91*
**Crus I VS IX**	*-9.3**	*< .001*	*2.08*	*9.41**	*< .001*	*2.11*
**Crus I VS X**	*8.5**	*< .001*	*1.9*	1.88	> .9	0.42
**Crus II VS III**	*8.2**	*< .001*	*1.82*	-2.54	.725	0.57
**Crus II VS IV/V**	*7**	*< .001*	*1.56*	-3.08	0.222	0.7
**Crus II VS VI**	*4.4**	*.012*	*0.98*	-2.57	0.67	0.58
**Crus II VS VIIb**	*-7.7**	*< .001*	*1.71*	-1.9	> .9	0.43
**Crus II VS VIII**	*-4.9**	*.004*	*1.08*	2.2	> .9	0.49
**Crus II VS IX**	*-5.6**	*.001*	*1.26*	*5.29**	*.002*	*1.18*
**Crus II VS X**	*7.3**	*< .001*	*1.63*	-0.19	> .9	0.04
**III VS IV/V**	*-6.1**	*< .001*	*1.36*	-0.81	> .9	0.18
**III VS VI**	*-9.8**	*< .001*	*2.18*	1.34	> .9	0.3
**III VS VIIb**	*-10.8**	*< .001*	*2.42*	1.35	> .9	0.3
**III VS VIII**	*-19.3**	*< .001*	*4.33*	*3.97**	*.03*	*0.89*
**III VS IX**	*-29.6**	*< .001*	*6.62*	*12.04**	*< .001*	*2.69*
**III VS X**	-0.3	> .9	0.06	3.1	.213	0.69
**IV/V VS VI**	*-10**	*< .001*	*2.23*	2.18	> .9	0.49
**IV/V VS VIIb**	*-10**	*< .001*	*2.17*	1.7	> .9	0.38
**IV/V VS VIII**	*-17.6**	*< .001*	*4*	*4.55**	*.008*	*1.02*
**IV/V VS IX**	*-27.3**	*< .001*	*6.1*	*13.17**	*< .001*	*2.94*
**IV/V VS X**	3.4	.117	0.75	3.48	.089	0.78
**VI VS VIIb**	*-7.4**	*< .001*	*1.65*	0.91	> .9	0.2
**VI VS VIII**	*-13.2**	*< .001*	*2.95*	*3.79**	*.045*	*0.85*
**VI VS IX**	*-16**	*< .001*	*3.58*	*11.3**	*< .001*	*2.53*
**VI VS X**	*8.1**	*< .001*	*1.81*	1.81	> .9	0.4
**VIIb VS VIII**	-1.6	> .9	0.35	*4.37**	*.012*	*0.98*
**VIIb VS IX**	-2.7	.474	0.61	*5.4**	*.001*	*1.21*
**VIIb VS X**	*9.3**	*< .001*	*2.07*	0.46	> .9	0.1
**VIII VS IX**	-3	.289	0.66	*4.49**	*.009*	*1*
**VIII VS X**	*15**	*< .001*	*3.36*	-1.59	> .9	0.36
**IX VS X**	*19.2**	*< .001*	*4.28*	*-6.43**	*< .001*	*1.44*

Supplementary Table S6. Descriptive electric field magnitudes for non-lateralized cerebellar vermis regions. Means ± standard deviations are shown, according to ROIs of the AAL atlas.

	$\|E\|_{target}$ [V/m]
**Vermis I/II**	0.07 ± 0.013
**Vermis III**	0.07 ± 0.008
**Vermis IV/V**	0.1 ± 0.01
**Vermis VI**	0.11 ± 0.014
**Vermis VII**	0.12 ± 0.018
**Vermis VIII**	0.12 ± 0.017
**Vermis IX**	0.13 ± 0.013
**Vermis X**	0.11 ± 0.013
